# Academic Publication of Anesthesiology From a Bibliographic Perspective From 1999 to 2018: Comparative Analysis Using Subject-Field Dataset and Department Dataset

**DOI:** 10.3389/fmed.2021.658833

**Published:** 2021-09-29

**Authors:** Sy-Yuan Chen, Ling-Fang Wei, Mu-Hsuan Huang, Chiu-Ming Ho

**Affiliations:** ^1^Division of Urology, Department of Surgery, Chang Gung Memorial Hospital-Linkou Branch, Taoyuan, Taiwan; ^2^National Central Library, Taipei, Taiwan; ^3^Department of Library and Information Science, National Taiwan University, Taipei, Taiwan; ^4^Department of Anesthesiology, Taipei Veterans General Hospital and National Yang Ming Chiao Tung University, Taipei, Taiwan

**Keywords:** anesthesia, anesthesiology, bibliometrics, informetrics, research evaluation, scientometrics

## Abstract

**Background:** Publication activity in the field of anesthesiology informs decisions that enhance academic advancement. Most previous bibliometric studies on anesthesiology examined data limited to journals focused on anesthesiology rather than data answerable to authors in anesthesia departments. This study comprehensively explored publication trends in the field of anesthesiology and their impact. We hypothesized that anesthesiology's bibliometric scene would differ based on whether articles in the same study period were published in anesthesiology-focused journals or were produced by authors in anesthesia departments but published in non-specialty journals.

**Methods:** This cross-sectional study used bibliometric data from the Science Citation Index Expanded database between 1999 and 2018. Two datasets were assembled. The first dataset was a subject-dataset (articles published in 31 journals in the anesthesiology category of InCites Journal Citation Reports in 2018); the second dataset was the department-dataset (articles published in the Science Citation Index Expanded by authors in anesthesia departments). We captured the bibliographical record of each article in both datasets and noted each article's Institute for Scientific Information code, publication year, title, abstract, author addresses, subject category, and references for further study.

**Results:** A total of 69,593 articles were published—cited 1,497,932 times—in the subject-dataset; a total of 167,501 articles were published—cited 3,731,540 times—in the department-dataset. The results demonstrate differences between the two datasets. First, the number of articles was stagnant, with little growth (average annual growth rate = 0.31%) in the subject-dataset; whereas there was stable growth (average annual growth rate = 4.50%) in articles in the department-dataset. Second, only 30.4% of anesthesia department articles were published in anesthesiology journals. Third, journals related to “pain” had the lowest department-subject ratio, which was attributable to a large portion of non-anesthesia department researchers' participation in related research.

**Conclusions:** This study showed that articles published in anesthesiology-focused and non-specialty journals demonstrate fundamentally different trends. Thus, it not only helps researchers develop a more comprehensive understanding of the current publication status and trends in anesthesiology, but also provides a basis for national academic organizations to frame relevant anesthesiology development policies and rationalize resource allocation.

## Introduction

Academic publications are not only the transmission of scientific knowledge but also the presentation of research achievements. Bibliometrics includes the application of mathematical and statistical methods to academic publications, and allows researchers to decipher the current research activities within a specific academic discipline (1–3). With the recent developments in anesthesia practices and the increasing endeavors of scientific research, anesthesia studies and their clinical applications have made great strides, which invites further research on anesthesiology-related bibliometric studies. In recent years, there have been a number of bibliometric studies on anesthesiology, focused on the investigation of research publication and impact in different countries (4–7), specific institutes (7–9), and individual authors (10, 11).

Notably, most bibliometric studies on anesthesiology examined data are only limited to anesthesiology journals, not all those produced by authors in anesthesia departments. Previous studies limited to anesthesiology journals reported the decreasing global publication of work on anesthesiology (6), including in the United States (12, 13), European countries (14), and the United Kingdom (15). However, this is difficult to quantify (16, 17), because many influential and highly cited anesthesiology-related papers are published in non-anesthesiology journals. Thus, the data scope of bibliometric studies on anesthesiology should be expanded from the subject-field journals to the authors' affiliated departments.

Research activity of anesthesiology includes both clinical and basic studies. Distinct from the aforementioned studies on anesthesiology conducted on a subject-field basis, there were a few bibliometric studies on global clinical trials (18), as well as publication of clinical research from the G-20 countries (19), the European countries (20, 21), Scandinavian countries (22), Canada (23), and East Asia (24), based on authors' affiliated departments. It is important to also inspect comprehensive department-based data, including all clinical and basic studies, in order to more thoroughly understand the overall condition of publications dealing with anesthesiology.

To date, there have been no formal bibliometric studies on global publication that include clinical and basic studies, nor focusing on the impact of anesthesiology in both the subject-field dataset and the researchers' affiliated department dataset. Hence, we hypothesized that the bibliometric scene is different between the subject-field dataset and department dataset in the same study period. We decided to conduct this study to observe global trends in publication and the impact of both the subject-field dataset and department dataset on anesthesiology in the period 1999–2018.

## Materials and Methods

### Study Design

This study was conducted in line with the ethical principles for medical research according to the Declaration of Helsinki. No ethics committee approval was required for this bibliographic study, because the data were obtained from the public domain of the National Taiwan University Library website on September 30, 2019. Data analyses were conducted from October 1, 2019, through December 31, 2019. We observed the Strengthening the Reporting of Observational Studies in Epidemiology (STROBE) reporting guidelines for cross-sectional studies.

### Bibliometric Approach

**Figure 1:**
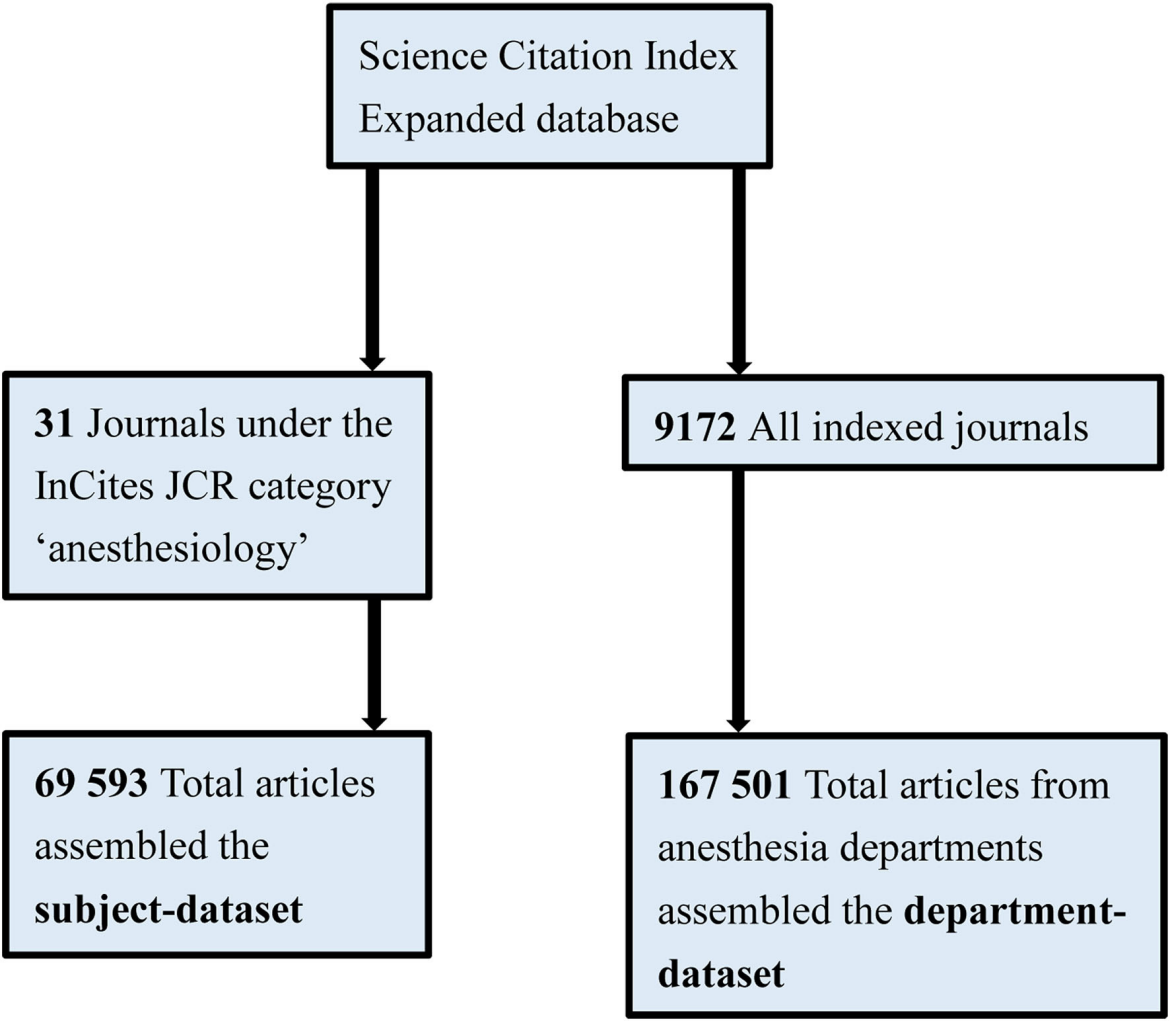
Study flow diagram.

Our study conducted a bibliometric analysis and collected anesthesiology bibliographic data across 20 years, from 1999 to 2018 (Figure 1). To achieve its research purpose, this study established two anesthesiology datasets: the subject-field dataset [papers from journals indexed in the subject-field according to the InCites Journal Citation Reports (JCR) category], and the department dataset [papers published in the names of departments from the Web of Science: Science Citation Index Expanded (SCIE) database]. For the former, we collected papers from 31 journals under the JCR category “anesthesiology” in 2018. We searched the journal names on the SCIE database and obtained the papers. For the latter, we defined anesthesia departments as academic groups or organizations that perform anesthesiology-related practices; therefore, these department papers referred to those published by clinicians and related researchers. In order to maximize the complete download of the global anesthesia department output, we use keywords in seven languages (English, Dutch, French, German, Italian, Portuguese, and Spanish) to conduct our search using authors' affiliation-addresses present in the SCIE database, and the represented departments which published papers were analyzed. Based on the research on the history of anesthesiology, the classical use of anaesthesiology (US: anesthesiology) or anaesthesia (US: anesthesia) to describe the specialty of anesthesiologists (25, 26); so we used anesthes^*^, anaesthes^*^, anesthés^*^, anästhes^*^, and anestes^*^ as the keywords for search. The symbol “^*^” is a placeholder for retrieving all search items that start with the preceding text. This may maximize the access to papers published by authors in the anesthesia department.

Only the document type “article” was analyzed in this study. “Article” was understood as a report of original research (27), and included research papers, brief communications, case reports, and technical notes. We noted the bibliographical data of each article in both datasets and recorded each article's Institute for Scientific Information code, publication year, title, abstract, author addresses, subject category, and references for advanced study.

As with our previous bibliometric studies (7, 28), the whole counting method (29), in which each co-author and publication were counted once, were used in this study. For the counting of research field, there were a total of 236 subject fields in the 2018 JCR, and each article in the SCIE database would be assigned to at least one subject field. In order to identify the proportion of anesthesiology publications among the different subject fields, this study adopted a specific classification and counting method, that is, anesthesiology = 1; other fields (non-anesthesiology) = 0. For example, when Paper X was assigned to “anesthesiology” and “Field A,” then this article would be coded as 1, and “Field A” would not be coded. By comparison, when Paper Y was assigned to “Field B” and “Field C,” then this article would be coded as 0.

### Statistical Analysis

Through calculating the number of articles and their times of citation, this study analyzed the publication trend and impact of both the subject-field and department datasets. To further evaluate the differences in terms of research publication between the two datasets, we calculated a “department-field ratio” using the following formula:

$$\begin{array}{l} \text{department}-\text{field ratio}=\frac{JD/WD\;}{JF/WF} \end{array}$$

^*^JD = the number of articles of a journal's department; WD = the number of articles of the departments worldwide; JF = the number of articles of a journal's subject-field; WF = the number of articles of the subject-field worldwide.

The department-field ratio can help us understand the difference between the department dataset and the subject-field dataset in terms of their number of articles within the same journal. A higher department-field ratio implies a smaller difference between the two datasets, whereas a smaller ratio indicates a greater difference.

Because our objective was only to describe the data of the two datasets, descriptive statistics were used.

## Results

### Overall Number of Articles

According to the subject-field dataset, there were 69,593 articles published from 1999 to 2018, with an average of 3,480 articles per year. Since the total number of articles published in the first 10 years made up 48.7% of the overall amount, it can be concluded that the remaining 10 years of the period exhibited a weak growth (2.56%). The number of articles per year from 1999 to 2008 grew from 3,346 to 3,625, achieving an average annual growth rate of 0.91%; meanwhile, publications from 2009 to 2018 dropped from 3,618 to 3,504, resulting in a negative average annual growth rate of −0.25%.

In the department dataset, there were 167,501 articles published from 1998 to 2018, with an average of 8,375 articles per year. Within that period, there was steady growth; the first 10 years (1999–2008) demonstrated a stable rise, and the remaining 10 years (2009–2018) showed a robust growth, which was corroborated when the annual number of articles exceeded 10,000 by 2014. Moreover, the 3 year-long periods with the highest annual growth rates were all included in the second half of the 20 years, which were 2010–2011 (8.88%), 2015–2016 (8.82%), and 2012–2013 (8.23%).

**Figure 2:**
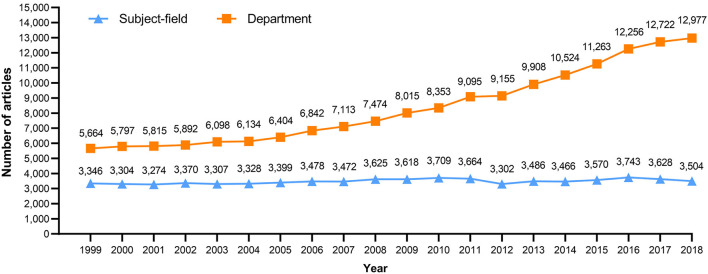
Trend of articles published in the subject-field dataset and the department dataset from 1999 to 2018.

The differences in annual number of articles between the subject-field dataset and the department dataset throughout the 20 years are reflected in Figure 2. It can be seen that the total number of articles published by authors within anesthesia departments was 2.4 times that of the subject-field. The average annual growth rate from 1999 to 2018 of the subject-field was around 0.31%, whereas that of the department was 4.50%.

### Analysis and Comparison of the Two Datasets

**Figure 3:**
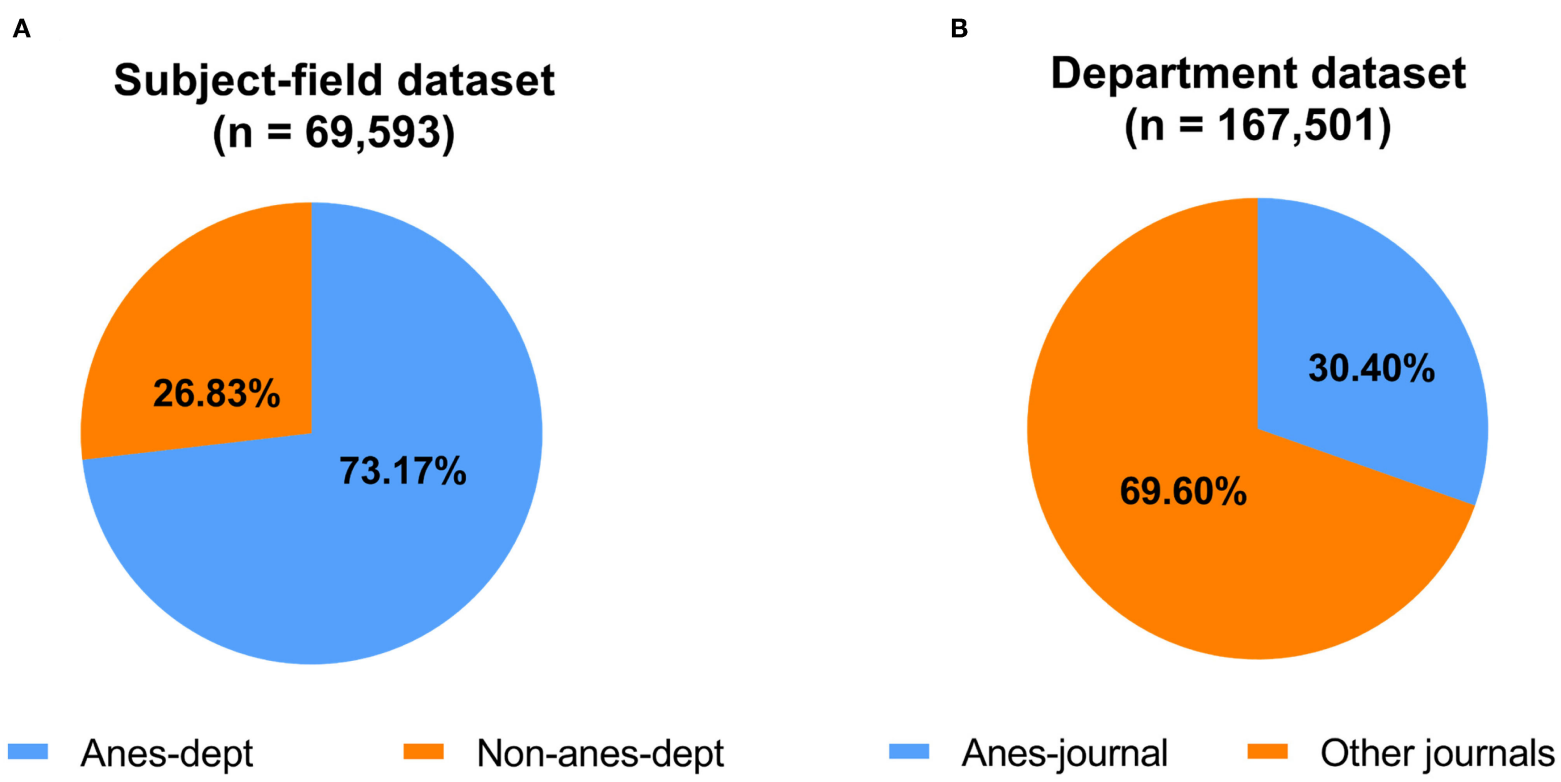
Percentage distribution of articles. **(A)** Authors of articles in the subject-field dataset from anesthesia departments (anes-dept) and non-anesthesia departments (non-anes-dept) from 1999 to 2018. The size of the circles represents the total number (*n*) of published articles. **(B)** Fields of publication in the department dataset from 1999 to 2018. The size of the circles represents the total number (*n*) of published articles from anesthesiology journals (anes-journals) and other journals.

By comparing the subject-field dataset with the department dataset, Figure 3A presents the affiliations of authors in anesthesiology. It shows that anesthesia department researchers were the main contributors to publications; 73.2% of the subject-field articles were produced by them and 26.8% of articles were produced by authors who belonged to the other domains.

The department dataset manifested the proportion of anesthesia department researchers who published articles in anesthesiology. Figure 3B shows that only 30.4% of anesthesia department articles were published by journals under the anesthesiology category in JCR 2018, while more of them (69.6%) were published by journals of other fields.

### Research Publication

Table 1 lists the top 20 journals with the most articles from the subject-field dataset, with *Anesthesia and Analgesia* (12.9%), *Anesthesiology* (7.9%), and *Pain* (7.2%) as the top three journals. These three journals accounted for 28.0% of the total, substantiating that little more than a quarter of the articles were published by them.

**Table 1:** The top 20 journals by number of articles from the subject-field dataset and department dataset in 1999–2018.

**Journals**	**Subject-field**		**Department**		**IF Rank**	**Dep.-field ratio**	Non-anes-fld.^*^
	**Article**	**Rank**	**Article**	**Rank**			
Anesthesia and Analgesia	8,986	1	8,298	1	8/31	0.38	–
Anesthesiology	5,491	2	4,835	2	1/31	0.37	–
Pain	5,026	3	1,132	23	3/31	0.09	–
British Journal of Anaesthesia	4,340	4	3,649	3	2/31	0.35	–
Journal of Cardiothoracic and Vascular Anesthesia	3,443	5	3,065	4	23/31	0.37	–
Acta Anaesthesiologica Scandinavica	3,304	6	2,915	5	18/31	0.37	–
Anaesthesia	2,873	7	1,955	10	4/31	0.28	–
Pediatric Anesthesia	2,588	8	2,241	8	21/31	0.36	–
Canadian Journal of Anaesthesia	2,325	9	2,122	9	9/31	0.38	–
Pain Medicine	2,231	10	723	29	15/31	0.13	–
Anaesthesia Critical Care and Pain Medicine	2,219	11	1,462	14	16/31	0.27	–
Anaesthesist	2,184	12	1,894	12	29/31	0.36	–
European Journal of Anaesthesiology	2,159	13	1,931	11	6/31	0.37	–
European Journal of Pain	2,156	14	412	56	10/31	0.08	–
Anaesthesia and Intensive Care	2,056	15	1,301	19	26/31	0.26	–
Journal of Clinical Anesthesia	1,948	16	1,814	13	7/31	0.39	–
Clinical Journal of Pain	1,828	17	511	49	13/31	0.12	–
Anasthesiologie Intensivmedizin Notfallmedizin Schmerztherapie	1,750	18	1,320	18	31/31	0.31	–
Regional Anesthesia and Pain Medicine	1,558	19	1,421	17	5/31	0.38	–
Journal of Anesthesia	1,541	20	1,432	16	25/31	0.39	–
PLoS ONE	–	–	2,340	6	24/69		MS
Critical Care Medicine	–	–	2,307	7	5/33		CCM
Intensive Care Medicine	–	–	1,437	15	2/33		CCM
Annals of Thoracic Surgery	–	–	1,221	20	45/136		CCS; RS; S
**Worldwide**	**69,593**	–	**167,501**	–			–

* *CCM, Critical Care Medicine; CCS, Cardiac and Cardiovascular Systems; MS, Multidisciplinary Sciences; RS, Respiratory System; S, Surgery*.

In the case of the department dataset, *Anesthesia and Analgesia* included the highest number of articles (4.9%), with the two next highest being *Anesthesiology* (2.9%) and the *British Journal of Anesthesia Anaesthesia* (2.3%). The articles of the anesthesia department were more dispersed, with 167,501 articles published in 4,813 journals, and they were more involved with cross-field and non-anesthesiology field journals, such as *PLoS ONE* (the field of multidisciplinary sciences), *Critical Care Medicine* (the field of critical care medicine), *Intensive Care Medicine* (the field of critical care medicine), and *Annals of Thoracic Surgery* (the fields of cardiac, cardiovascular, and respiratory systems, and surgery).

### Research Impact

Table 2 enumerates the journals that accumulated the most citation counts in the subject-field dataset from 1999 to 2018. In this dataset, the top four journals with the most citations also occupied the top four places by number of article published, though in a different order; this means, as far as the indicators of “research publication” and “research impact,” that they were the leading journals in the field, with an outstanding number of articles and influence.

**Table 2:** The top 20 journals by number of citations in the subject-field dataset and department dataset in 1999–2018.

**Journals**	**Subject-field**		**Department**		**IF rank**	**2018 IF**	Non-anes-Fld.^*^
	**Citation**	**Rank**	**Citation**	**Rank**			
Pain	285,260	1	61,087	7	3/31	6.03	–
Anesthesia and Analgesia	239,084	2	223,726	1	8/31	3.49	–
Anesthesiology	229,407	3	201,783	2	1/31	6.42	–
British Journal of Anaesthesia	131,524	4	110,825	4	2/31	6.20	–
Acta Anaesthesiologica Scandinavica	59,597	5	53,901	8	18/31	2.23	–
Anaesthesia	57,497	6	38,115	15	4/31	5.88	–
European Journal of Pain	54,758	7	11,323	56	10/31	3.19	–
Clinical Journal of Pain	51,935	8	12,579	46	13/31	2.89	–
Canadian Journal of Anaesthesia	39,540	9	37,023	17	9/31	3.37	–
Pediatric Anesthesia	37,702	10	33,504	21	21/31	2.04	–
Pain Medicine	37,415	11	11,172	58	15/31	2.76	–
Regional Anesthesia and Pain Medicine	35,301	12	32,784	22	5/31	5.11	–
Journal of Cardiothoracic and Vascular Anesthesia	33,332	13	29,579	24	23/31	1.88	–
European Journal of Anaesthesiology	31,694	14	28,525	25	6/31	4.14	–
Journal of Clinical Anesthesia	21,465	15	20,123	28	7/31	3.54	–
Anaesthesia and Intensive Care	20,048	16	13,399	45	26/31	1.36	–
Current Opinion in Anesthesiology	15,451	17	12,322	48	20/31	2.10	–
Pain Physician	14,460	18	6,213	100	12/31	2.94	–
Anaesthesist	12,935	19	12,058	52	29/31	0.90	–
Journal of Neurosurgical Anesthesiology	12,360	20	9,946	61	11/31	2.96	–
Critical Care Medicine	–	–	113,439	3	5/33	6.97	CCM
Intensive Care Medicine	–	–	65,429	5	2/33	18.97	CCM
Lancet	–	–	63,693	6	2/160	59.10	GIM
Journal of Neuroscience	–	–	53,130	9	29/267	6.07	N
Proceedings of the National Academy of Sciences of the United States of America	–	–	51,694	10	7/69	9.58	MS
New England Journal of Medicine	–	–	49,592	11	1/160	70.67	GIM
JAMA-Journal of the American Medical Association	–	–	47,358	12	3/160	51.27	GIM
Critical Care	–	–	39,639	13	6/33	6.96	CCM
Circulation	–	–	39,069	14	2/136	23.05	CCS; PVD
American Journal of Respiratory and Critical Care Medicine	–	–	37,603	16	3/33	16.49	CCM; RS
Annals of Thoracic Surgery	–	–	34,981	18	45/136	3.92	CCS; RS; S
Journal of Biological Chemistry	–	–	34,929	19	81/299	4.01	BMB
PLoS ONE	–	–	34,523	20	24/69	2.76	MS
**Worldwide**	**1,497,932**	–	**3,731,540**	–		–	–

* *BMB, Biochemistry and Molecular Biology; CCM, Critical Care Medicine; CCS, Cardiac and Cardiovascular Systems; GIM, General and Internal Medicine; MS, Multidisciplinary Sciences; N, Neurosciences; PVD, Peripheral Vascular Disease; RS, Respiratory System; S, Surgery*.

According to Table 2, there were 13 journals in the department dataset of top 20 journals citations that belonged to a non-anesthesiology subject, and these can be either divided or overlapped onto nine other medical subjects. The above phenomenon indicates a wide range of diversity that anesthesia department articles exhibited. It is noteworthy that some articles produced by the anesthesia department were published in prestigious journals in the general and internal medicine subjects, such as *New England Journal of Medicine* (IF = 70.67), *Lancet* (IF = 59.10), and *JAMA* (IF = 51.27), conveying that those articles, too, held a considerably high academic influence.

## Discussion

To our best knowledge, this is the first bibliometric anesthesiology study that synchronously analyzed articles in the subject-field dataset and the department dataset, covering data that spanned a long period of two decades. The results demonstrated some differences between these two datasets, presented in terms of the real academic publication activity in anesthesiology.

First, the present study illustrates a steady trend in the number of articles in the subject-field dataset and a stable growth in publication is observed in the department dataset across our observation period (1998–2018) (Figure 2). In some previous bibliometric studies, data from anesthesiology journals focused on clinical studies and reported a steady trend in publications before 2004 (6, 13–15) and a decrease in publications after 2004 (6, 14, 15). This phenomenon of a decrease in publications was not seen in the subject-field dataset of our study, which is consistent with our previous finding (7). This discrepancy may be a result of the inclusion in our study datasets of both clinical and non-clinical studies. Moreover, we showed that the number of publications from global anesthesia departments had a steady trend before 2004, but had an average annual growth rate that increased from 2.02 to 5.53% after 2004 (Figure 2). These findings were similar to former bibliometric studies of clinical research from anesthesia departments of G-20 countries (19), European countries (20, 21), and Scandinavian countries (22). Together with the subject-field's trend, the widening gap of publications between the two datasets was unveiled. Thus, the steady trend in publication in the subject-field dataset might not accurately reflect the actual activity of academic publication. In fact, there was increasing research done by anesthesiologists and related researchers, a phenomenon which likely explains the discrepancy between the two datasets from 2009 to 2018.

Second, our results show that a majority of articles (73.27%) from the subject-field dataset were produced by anesthesia department researchers (Figure 3A). In contrast, articles from the department dataset produced by anesthesia department researchers accounted for only 30.4% articles published in the 31 journals in the subject-field dataset (Figure 3B). Interestingly, a previous bibliometric study similarly reported that only 18% of laboratory research articles using molecular biology techniques from anesthesia department were published in anesthesiology journals (30); 82% of the articles were published in other biomedical journals. We also found that many of the articles by anesthesia department researchers that had a high number of citations were mostly published in non-anesthesiology-field journals (Table 2). This may explain why previous studies found that the decreasing number of articles in anesthesiology-field journals (6, 12–15) might be due to the changing submission direction of some authors. Together, these findings imply that authors sent their articles to other high-impact medical journals instead of those in the anesthesiology subject-field.

Our data also support the possibility that the articles published by the anesthesia department researchers revealed a myriad of subjects (Tables 1, 2), including: biochemistry and molecular biology, critical care medicine, cardiac and cardiovascular systems, general and internal medicine, multidisciplinary sciences, neurosciences, peripheral vascular disease, respiratory system, and surgery. Among them, critical care medicine received the most publication, whereas general and internal medicine generated a remarkable number of high-impact articles. In fact, anesthesia department researchers tended to obtain a higher degree of clinical cross-discipline involvement (31). We believe that cross-discipline interaction is important to provide the best care and break the boundaries between disciplines, through research, to provide our patients with better clinical outcome.

Notably, among the top 20 journals in the subject-field dataset (Table 1), there were four journals with a department-field ratio lower than 0.2, indicating a low publication rate from anesthesia department researchers. All four journals were international journals focused on the topic “pain.” Of all the journals, *Pain* presented an obvious variation in its ranking; in the subject-field dataset, it accumulated 5,026 articles and ranked the third, but in the department dataset, there were only 1,132 articles written by anesthesia department researchers and it ranked the 23rd. From the department-field ratio of 0.09, we may suggest that despite the profuse number of articles from *Pain* included in the subject-field dataset, nearly three quarters of them were written by non-anesthesia department researchers. This phenomenon was also perceivable in the *European Journal of Pain* (0.08), the *Clinical Journal of Pain* (0.12), and *Pain Medicine* (0.13). This was attributable to non-anesthesia department researchers' participation in research concerning “pain,” a topic whose representative journals were mostly composed of articles about basic medical science (32).

Bibliometric studies based on data from only either the subject-field or the researchers' affiliations could not fully represent the publication trends and impact in anesthesiology, and it is possible that such studies would produce biased findings (16, 17). As it compares the two synchronous datasets over the recent 20-year period, our study not only can help researchers gain a more comprehensive understanding of the current status of publication trends in anesthesiology, but also provide the exploitation of data and the use of novel information for national academic organizations to formulate relevant policies and rationalize resource allocation for the development of anesthesiology. This will encourage anesthesiologists to enhance the quality of their research for the benefit of patients.

This study's findings and implications have some potential limitations. First, we included only articles from the Web of Science: SCIE database, not all existing databases, such as PubMed. This indicates that some research papers are not included in this study. Although the SCIE database has a slightly smaller collection of articles in the medical field than PubMed, the SCIE database includes journals in a wider field (33), which helps to provide a more complete picture of the published fields of authors in the anesthesia department. In fact, we found that a few articles were published in humanities and arts journals, this finding in our study not likely to be found in PubMed. Briefly, we consider our results valid because the SCIE database is well-established and was the only database that provided the affiliations of all authors of a particular article for this long study period (33, 34). Next, some anesthesia departments and intensive care departments may have recently been merged and renamed, thus becoming a department with a broader name that does not include “anesthesia,” such as a department of perioperative medicine. Thus, this study may have omitted some publications because, for example, our keywords for anesthesia departments were not listed in the affiliation field for the department of perioperative medicine; therefore, articles from this department were excluded. However, it is likely that the small number of articles produced by these few departments are not significant compared to the 167,501 articles in the department dataset of our study. Finally, because of the limitations of the database, only publications in English were included, meaning that articles written in non-English languages may not all be identified. Nevertheless, we believe that English has been the dominant language in medical science for a long time (35).

In conclusion, our results not only explored the publication trends and impact in anesthesiology during the two recent decades, but also provided a more objective view of anesthesiology research activity. It can be seen that the publication trends in the subject-field dataset were stagnant, with little growth; whereas there was continuous growth in the department-dataset. For anesthesia departments, it was observed that more of the articles were published in cross-discipline journals or those of other subject-fields. Furthermore, a large portion of highly-cited articles were published in high-profile non-anesthesiology journals, and our results reveal the submission targets of anesthesia researchers.

## Data Availability Statement

The datasets generated for this study can be found in online repositories. The names of the repository/repositories and accession number(s) can be found below: This cross-sectional study used bibliometric data from the Science Citation Index Expanded database between 1999 and 2018.

## Author Contributions

S-YC drafted the first draft. M-HH and C-MH revised the manuscript for important intellectual content, provided administrative and obtained funding. L-FW and M-HH provided specific expertise on bibliometrics and contributed to data management. C-MH supervised this study. All authors were involved in study conception and design as well as data acquisition, analysis, interpretation, and reviewed and approved the manuscript.

## Funding

This work was financially supported in part by the Ministry of Science and Technology (MOST), Taiwan, under Grant nos. MOST 106-2410-H-002-091-MY2, MOST 108-2314-B-075-025-MY2, MOST 110-2634-F-002-045, the Ministry of Education (MOE), Taiwan, under Grant nos. MOE 110L900204, MOE 110L9A002, and the Taipei Veterans General Hospital, Taiwan, under Grant no. V109C-167, V110C-146.

## Conflict of Interest

The authors declare that the research was conducted in the absence of any commercial or financial relationships that could be construed as a potential conflict of interest.

## Publisher's Note

All claims expressed in this article are solely those of the authors and do not necessarily represent those of their affiliated organizations, or those of the publisher, the editors and the reviewers. Any product that may be evaluated in this article, or claim that may be made by its manufacturer, is not guaranteed or endorsed by the publisher.
